# Supplementary material for: Host genetic factors related to innate immunity, environmental sensing and cellular functions are associated with human skin microbiota
Source: Nat Commun. 2022 Oct 19;13:6204. doi: 10.1038/s41467-022-33906-5 (PMC9582029; doi:10.1038/s41467-022-33906-5)
Supplement: Supplementary file 1 — Supplementary Information [file 41467_2022_33906_MOESM1_ESM.pdf]

Title

Host genetic factors related to innate immunity, environmental sensing and cellular functions are associated with human skin microbiota

Author list

Lucas Moitinho-Silva[1,2], Frauke Degenhardt[1], Elke Rodriguez[2], Hila Emmert[2], Simonas Juzenas[1,3], Lena Möbus[2], Florian Uellendahl-Werth[1], Nicole Sander[2], Hansjörg Baurecht[4], Lukas Tittmann[5], Wolfgang Lieb[6], Christian Gieger[7,8], Annette Peters[7], David Ellinghaus[1], Corinna Bang[1], Andre Franke[1#*], Stephan Weidinger[2#*] and Malte Christoph Rühlemann[1#]

Affiliations

[1]Institute of Clinical Molecular Biology, Kiel University, Kiel, Germany

[2]Department of Dermatology and Allergy, University Hospital Schleswig-Holstein, Kiel, Germany

[3]Institute of Biotechnology, Life Science Centre, Vilnius University, Vilnius, Lithuania

[4]Department for Epidemiology and Preventive Medicine, University of Regensburg, Regensburg, Germany

[5]Biobank PopGen and Institute of Epidemiology, Kiel University, Kiel, Germany

[6]Institute of Epidemiology, Kiel University, Kiel, Germany

[7]Institute of Epidemiology, Helmholtz Zentrum München – German Research Center for Environmental Health, Neuherberg, Germany

[8]Research Unit of Molecular Epidemiology, Helmholtz Zentrum München – German Research Center for Environmental Health, Neuherberg, Germany

[#]Authors contributed equally.

*Corresponding authors: AF, a.franke@mucosa.de; SW, sweidinger@dermatology.uni-kiel.de.

Supplementary Table

Supplementary Table 1. Samples collected for microbial profiling.

	KORA FF4 (N = 635)	PopGen (N = 1021)
**Dry**		
Dorsal forearm	-	260 (25)
Volar forearm	-	251 (25)
**Moist**		
Antecubital fossa	318 (50)	258 (25)
**Sebaceous**		
Forehead	-	252 (25)
Retroauricular fold	317 (50)	-

Percentages are shown (%).
