# Supplementary material for: Host genetic factors related to innate immunity, environmental sensing and cellular functions are associated with human skin microbiota
Source: Nat Commun. 2022 Oct 19;13:6204. doi: 10.1038/s41467-022-33906-5 (PMC9582029; doi:10.1038/s41467-022-33906-5)
Supplement: Supplementary file 3 — Description of Additional Supplementary Files [file 41467_2022_33906_MOESM3_ESM.pdf]

Description of Additional Supplementary Files

File Name: Supplementary Data 1
Description: Per data set results of GWAS for microbial features. Variants were selected based on loci fine mapping analysis (FM) or when variants had a linkage disequilibrium >0.6 (LD > 0.6). Single variant association tests were performed for each sample type and each microbial feature. Tests were adjusted for age, sex, body mass index (BMI) and genetic background (first ten genetic principal components). Positions are given as in genome assembly hg19 (GRCh37). Effect allele frequency (EAF) is indicated. Non-redundant microbial features and features which belongs to the same cluster are indicated. Microbial features are prefixed with their level, amplicon variant sequence (a.), genus (g.), family (f.), order (o.), class (c.) or phylum (p.). Test summaries, total sample number (N), effect size (β) and standard error (s.e.) and *P* value, are reported for tests data sets. Distance-based F-test using moment matching was performed for beta diversity and two-sided t-test was performed for univariate microbial features. Data sets 1 and 2 indicate each skin site and cohort combination per microenvironment as stated in “Data sets” column. Genes with variants within their region (no formatted font) or with variants associated with their expression (italic font) are shown. Genes are shown in bold font when both conditions are met. ENSG00000263272 is a novel transcript, antisense to RPAIN and ENSG00000269886 is a novel transcript, antisense to TTLL3.

File Name: Supplementary Data 2
Description: Genes found in association with skin microbial features.

File Name: Supplementary Data 3
Description: Differentially expressed genes in keratinocytes co-cultured with *S. epidermidis* in comparison with control. Transcripts that passed the significance cut-off are shown [*q* values < 0.05 (derived from Wald test on negative binomial generalized linear models) and absolute log2 fold change >1]. Approximate posterior estimation for generalized linear model (apeglm[76]) shrinkage was applied to log2 fold change

File Name: Supplementary Data 4
Description: Enriched GO biological processes based on up- and down-regulated genes in keratinocytes co-cultured with *S. epidermidis* in comparison with control. GO biological processes that passed the significance cut-off are shown (*q* values < 0.05; Fisher exact test) and ordered by the smallest *q* values.

File Name: Supplementary Data 5
Description: Mendelian randomization results. Non-infectious skin diseases were set as outcome and microbial features were set as exposure in inverse-variance-weighted analysis. Non-redundant microbial features and features which belongs to the same cluster are indicated. Microbial features are prefixed with their level, amplicon variant sequence (a.), genus (g.), family (f.), order (o.), class (c.) or phylum (p.). Mean and minimum (min.) F-statistics are shown. Tests were two-sided. Number of instruments (No. of SNPs), effect size (β) and standard error (s.e.) and *P* value from inverse variance weighted (IVW) MR or Wald-ratio (see Methods) are reported. False discovery rate (FDR) was applied per trait ($q_{(trait)}$) and for the total amount of tests ($q_{(global)}$). Significant *P* and *q* values are in bold font.
