# Supplementary material for: Host genetic factors related to innate immunity, environmental sensing and cellular functions are associated with human skin microbiota
Source: Nat Commun. 2022 Oct 19;13:6204. doi: 10.1038/s41467-022-33906-5 (PMC9582029; doi:10.1038/s41467-022-33906-5)
Supplement: Supplementary file 9 — Reporting Summary [file 41467_2022_33906_MOESM9_ESM.pdf]

nature portfolio

Corresponding author(s): Lucas Moitinho-Silva

Last updated by author(s): Sep 4, 2022

Reporting Summary

Nature Portfolio wishes to improve the reproducibility of the work that we publish. This form provides structure for consistency and transparency in reporting. For further information on Nature Portfolio policies, see our Editorial Policies and the Editorial Policy Checklist.

Statistics

For all statistical analyses, confirm that the following items are present in the figure legend, table legend, main text, or Methods section.

n/a	Confirmed	
☐	☒	The exact sample size (*n*) for each experimental group/condition, given as a discrete number and unit of measurement
☐	☒	A statement on whether measurements were taken from distinct samples or whether the same sample was measured repeatedly
☐	☒	The statistical test(s) used AND whether they are one- or two-sided<br>*Only common tests should be described solely by name; describe more complex techniques in the Methods section.*
☐	☒	A description of all covariates tested
☐	☒	A description of any assumptions or corrections, such as tests of normality and adjustment for multiple comparisons
☐	☒	A full description of the statistical parameters including central tendency (e.g. means) or other basic estimates (e.g. regression coefficient) AND variation (e.g. standard deviation) or associated estimates of uncertainty (e.g. confidence intervals)
☐	☒	For null hypothesis testing, the test statistic (e.g. *F*, *t*, *r*) with confidence intervals, effect sizes, degrees of freedom and *P* value noted<br>*Give P values as exact values whenever suitable.*
☒	☐	For Bayesian analysis, information on the choice of priors and Markov chain Monte Carlo settings
☒	☐	For hierarchical and complex designs, identification of the appropriate level for tests and full reporting of outcomes
☐	☒	Estimates of effect sizes (e.g. Cohen's *d*, Pearson's *r*), indicating how they were calculated

Our web collection on statistics for biologists contains articles on many of the points above.

Software and code

Policy information about availability of computer code

Data collection	No software was used for data collection.
Data analysis	The following software were used:<br>IKMB GWAS Quality Control Pipeline (https://github.com/ikmb/gwas-qc)<br>Michigan Imputation Server (https://imputationserver.sph.umich.edu/index.html)<br>miQTL cookbook (https://github.com/alexa-kur/miQTL_cookbook#chapter-2-genotype-imputation).<br>SHAPEIT v2<br>IMPUTE v2.3<br>PLINK v1.9<br>Genotype Harmonizer v 1.4.23<br>PLINK v2.0-alpha-avx2-20200217<br>R package DADA2 v1.10<br>R v3.6.2<br>R v3.6.1<br>R package Phyloseq v1.34.0<br>R package Vegan v2.5-5<br>R package Mvabund v4.1.6<br>R package GenABEL v1.8-0<br>METAL release 2011-03-25<br>METASOFT v2<br>FINEMAP v1.4

Human genome browser at the University of California Santa Cruz (UCSC) (10.1101/gr.229102
R package TwoSampleMR v0.5.5
nf-core/rnaseq pipeline v3.0
R package DESeq2 v.1.30.0
R package enrichR v3.0
R package PCAtools v. 2.4.0

For manuscripts utilizing custom algorithms or software that are central to the research but not yet described in published literature, software must be made available to editors and reviewers. We strongly encourage code deposition in a community repository (e.g. GitHub). See the Nature Portfolio guidelines for submitting code & software for further information.

Data

Policy information about availability of data

All manuscripts must include a data availability statement. This statement should provide the following information, where applicable:

- Accession codes, unique identifiers, or web links for publicly available datasets
- A description of any restrictions on data availability
- For clinical datasets or third party data, please ensure that the statement adheres to our policy

Raw 16S rRNA gene amplicon sequences of PopGen participants were deposited at the European nucleotide archive (ENA) under accession code PRJEB41215 [https://www.ebi.ac.uk/ena/browser/view/PRJEB41215?show=reads]. GWAS summary statistics generated in this study are available at GWAS catalogue under accession codes GCST90133164-GCST90133313. Phenotype data from PopGen individuals can be accessed through the Material Data Access Form from the PopGen Biobank (Schleswig-Holstein, Germany). Information about the Material Data Access Form and how to apply can be found at http://www.uksh.de/p2n/Information+for+Researchers.html. KORA data are available at https://www.helmholtz-munich.de/en/kora/for-scientists/cooperation-with-kora/index.html upon request by means of a project agreement.
In addition, the following public database and resources were used: 1000 Genomes Phase3 reference47, Ribosomal Database Project (RDP) version 1654, Genotype-Tissue Expression (GTEx) Project database v810, Functional annotation of the mammalian genome (FANTOM5)68, Skin single-cell data from by Solé-Boldo et al12, UK Biobank and the IEU Open GWAS Project database70.

Human research participants

Policy information about studies involving human research participants and Sex and Gender in Research.

Reporting on sex and gender	The biological attribute sex was used. It was self-reported as part of the data collection from cohorts PopGen and KORA FF4.
Population characteristics	Age distribution, BMI and sex frequencies are described in Figure 1.
Recruitment	For PopGen, participants (aged 19–77 years) were randomly recruited between 2005 and 2007, and reinvited in 2016–2017 (second follow-up) via the local population registry and as blood donors from the region of Kiel, Germany. KORA FF4 is the second follow-up of the KORA S4 Survey (1999–2001, aged 25–74 years) conducted between 2013 and 2014 in the southern German city of Augsburg and its two surrounding counties. Participants of KORA S4 Survey were randomly selected.
Ethics oversight	All protocols were approved by the ethics committees of the Medical Faculty of Kiel University (PopGen) and of the Bavarian Medical Association (KORA).

Note that full information on the approval of the study protocol must also be provided in the manuscript.

Field-specific reporting

Please select the one below that is the best fit for your research. If you are not sure, read the appropriate sections before making your selection.

☒ Life sciences ☐ Behavioural & social sciences ☐ Ecological, evolutionary & environmental sciences

For a reference copy of the document with all sections, see nature.com/documents/nr-reporting-summary-flat.pdf

Life sciences study design

All studies must disclose on these points even when the disclosure is negative.

Sample size	A total of 1,656 skin samples from participants of two cross-sectional, population-based German cohorts, KORA FF4 (individuals=324) and PopGen (individuals=273) were analyzed. Sample sizes were limited data availability, i.e., we took all samples available in PopGen and KORA FF4. While higher sample size would result in higher discovery power, this sample size was useful for our study purpose which was to discovery associtations between the host genetic repertoire and the skin microbiome. For keratinocyte cell cultures, six replicates were performed per condition. Sample size was chosen based on the other publications which cultures Staphylococcus species with keratinocytes (e.g., Sayedyahossein et al. 2015 (DOI: 10.1096/fj.14-262774))
Data exclusions	Variants which did not passed the quality control were excluded from the analyses.

Replication	GWAS findings based were based on tow cohorts which were combined via meta-analysis. All attempts at replication were successfully. Keratinocyte cell cultures, six replicates were performed per condition, in three batches of two replicates per week. All attempts at replication were successfully.
Randomization	Covariates like sequencing batch, sex, age and BMI were controlled during statistical modelling when appropriate.
Blinding	Blinding is not relevant to this study because this is a population-based survey.

Reporting for specific materials, systems and methods

We require information from authors about some types of materials, experimental systems and methods used in many studies. Here, indicate whether each material, system or method listed is relevant to your study. If you are not sure if a list item applies to your research, read the appropriate section before selecting a response.

Materials & experimental systems

n/a	Involved in the study
☒	☐ Antibodies
☐	☒ Eukaryotic cell lines
☒	☐ Palaeontology and archaeology
☒	☐ Animals and other organisms
☒	☐ Clinical data
☒	☐ Dual use research of concern

Methods

n/a	Involved in the study
☒	☐ ChIP-seq
☒	☐ Flow cytometry
☒	☐ MRI-based neuroimaging

Eukaryotic cell lines

Policy information about cell lines and Sex and Gender in Research

Cell line source(s)	Normal human epidermal keratinocytes (NHEKs) (foreskin of a 0-year-old male Caucasian donor; Promocell, Heidelberg, Germany, Lot number 407Z001)
Authentication	Cells were phenotypically characterized based on cytokeratin tested with Flow cytometry as informed by the provider.
Mycoplasma contamination	PCR negative for Mycoplasma as informed by provider.
Commonly misidentified lines (See ICLAC register)	-
